# Brain Natriuretic Peptide for Predicting Contrast-Induced Acute Kidney Injury in Patients with Acute Coronary Syndrome Undergoing Coronary Angiography: A Systematic Review and Meta-Analysis

**DOI:** 10.1155/2020/1035089

**Published:** 2020-09-19

**Authors:** Xiaoming Li, Chao Liu, Zhi Mao, Shuang Qi, Renjie Song, Feihu Zhou

**Affiliations:** ^1^Medical School of Chinese PLA, Beijing, China; ^2^Department of Critical Care Medicine, The First Medical Centre, Chinese PLA General Hospital, Beijing, China

## Abstract

**Objective:**

To assess the diagnostic value of B-type natriuretic peptide (BNP) or N-terminal pro-B-type natriuretic peptide (NT-proBNP) for contrast-induced acute kidney injury (CI-AKI) in patients with acute coronary syndrome (ACS) undergoing coronary angiography.

**Background:**

ACS remains a major cause of death worldwide. Patients with ACS undergoing coronary angiography are more likely to develop CI-AKI, which correlates highly with poor clinical outcomes. Early diagnosis of CI-AKI remains a challenge. Many recent studies have suggested that BNP or NT-proBNP may be a useful biomarker for the early diagnosis of CI-AKI.

**Methods:**

We searched databases (PubMed, EMBASE, and Cochrane Library) to identify eligible studies. Two authors independently screened the studies and extracted data. We used the Quality Assessment of Diagnostic Accuracy Studies 2 (QUADAS-2) criteria to assess the methodological quality of the included studies and STATA to perform all statistical analyses.

**Results:**

Nine studies including 2832 patients were identified. The pooled sensitivity of 0.73 (95% CI 0.65–0.79), specificity of 0.79 (95% CI 0.70–0.85), and area under the summary receiver operating characteristic curve of 0.81 (95% CI 0.77–0.84) suggested that BNP or NT-proBNP had a good diagnostic value for CI-AKI in patients with ACS undergoing coronary angiography.

**Conclusions:**

Our findings suggest that BNP or NT-proBNP may be an effective predictive marker for CI-AKI. However, additional high-quality studies are required to find the optimal cutoff value and the diagnostic value of BNP or NT-proBNP in combination with other biomarkers.

## 1. Introduction

Acute coronary syndrome (ACS), including unstable angina (UA), non-ST elevation myocardial infarction (NSTEMI), and ST-elevation myocardial infarction (STEMI), generally results from atherosclerotic plaque rupture or superficial plaque erosion [1, 2]. Despite great progress in the treatment of ACS over the past few decades, ACS is still a major cause of death worldwide [3]. For patients with ACS, coronary angiography plays a key role. Early invasive treatment with cardiac catheterization and revascularization remains the preferred treatment for UA and NSTEMI, and timely percutaneous coronary intervention (PCI) for STEMI is recommended as a first-line treatment when prohibitive comorbidities are absent [4–6]. These treatments can reduce mortality and improve prognosis in patients with ACS.

Acute kidney injury (AKI) is a common and serious complication of inpatients that causes significant mortality and other severe complications [7, 8]. Patients with ACS, especially those undergoing coronary angiography or PCI, are more likely to develop AKI due to contrast agent exposure [9, 10]. The development of CI-AKI after coronary angiography is highly correlated with poor clinical outcomes, such as mortality [11–13], adverse cardiac events [14], and stent restenosis [15]. The ability to identify patients at high risk for developing CI-AKI identified early is important to allow the treating physician to take necessary precautions to prevent it.

Brain natriuretic peptides are released into the circulation in response to myocardial ischemia, pressure overload, or ventricular dilatation [16, 17]. Previous studies have found elevated concentrations of B-type natriuretic peptide (BNP) or N-terminal pro-B-type natriuretic peptide (NT-proBNP) in patients with ACS and have a prognostic value in patients with ACS [18, 19]. Moreover, some studies have found that levels of BNP or NT-proBNP are higher in patients with AKI [20–22], especially for those who are diagnosed with ACS and undergo coronary angiography or PCI [23–25].

To fully understand the correlation between elevated levels of brain natriuretic peptide and CI-AKI, we performed this meta-analysis to evaluate the diagnostic value of brain natriuretic peptide for CI-AKI in patients with ACS undergoing coronary angiography.

## 2. Methods

We conducted this meta-analysis following the Preferred Reporting Items for Systematic Reviews and Meta-Analyses (PRISMA statement) guidelines [26]. There was no prospectively registered protocol; however, search terms, data extraction, inclusion and exclusion criteria, and data synthesis were applied according to a plan made by our team.

### 2.1. Selection of Studies

We reviewed PubMed, EMBASE, and the Cochrane Central Register of Controlled Trials Library database through April 2020. The search terms were as follows: (“B-type natriuretic peptide” or “BNP” or “N-terminal pro-B-type natriuretic peptide” or “NT-proBNP”) and (“acute kidney injury” or “AKI” or “contrast-induced acute kidney injury” or “CI-AKI” or “contrast-induced nephropathy” or “CIN”) and (“acute coronary syndrome” or “ACS” or “acute myocardial infarction” or “AMI” or “ST-elevation myocardial infarction” or “STEMI” or “Non–ST elevation myocardial infarction” or “NSTEMI” or “unstable angina”). We did not impose any language restrictions. To find additional citations, the reference lists of the included studies and recent reviews were manually searched when necessary.

Studies were selected if they met the following criteria: a diagnostic value of BNP or NT-proBNP for CI-AKI morbidity in adult patients (≥18 years old) with ACS undergoing coronary angiography or PCI was reported; a 2 × 2 table of results could be constructed; CI-AKI was clearly defined; and the study type was a prospective or retrospective study. The exclusion criteria were as follows: case report, review, editorial, conference abstract, comment, letter, animal study, involving pediatric patients, and insufficient information to extract a 2 × 2 table of results. Two authors (X. L and C. L) assessed the selected studies for the final analysis independently, and any discrepancies were resolved through consultation with the third author (F. Z).

### 2.2. Data Extraction and Quality Assessment

The following data were extracted by two authors (X. L and C. L) and checked by the third author (Z. M): the first author, year of publication, study design, sample size, average age, patient population, definition of CI-AKI, measurement method of brain natriuretic peptide, timing of brain natriuretic peptide measurement, cutoff points, area under the curve (AUC), true positives (TP), true negatives (TN), false positives (FP), false negatives (FN), sensitivity (SEN), and specificity (SPE).

We used the QUADAS-2 (Quality Assessment of Diagnostic Accuracy Studies-2) criteria to evaluate each of these studies in 4 domains: patient selection; index test; reference standard; and flow and test timing [27]. Any disagreements were resolved by discussion.

### 2.3. Statistical Analysis

The statistical analyses were conducted by STATA (version 14.0) using the MIDAS module [28]. A bivariate random-effects regression model was performed to calculate SEN, SPE, the positive likelihood ratio (PLR), the negative likelihood ratio (NLR), the diagnostic odds ratio (DOR), and the corresponding 95% credible interval (CI). A summary receiver operating characteristic (SROC) curve was drawn to assess the overall diagnostic accuracy [29]. We used the Deek funnel plots to detect publication bias, whereby publication bias may exist if the *P* value is less than 0.1. The *I*^2^ index was calculated to assess heterogeneity between studies, and *I*^2^ values above 50% were regarded as indicative of substantial heterogeneity. We generated a Fagan nomograph and likelihood ratio scattergram to evaluate clinical applications. Sensitivity and subgroup analyses were conducted to investigate potential sources of heterogeneity among the included studies.

## 3. Results

### 3.1. Selection and Characteristics of Studies

As a result of the literature search, 170 studies were identified, of which 55 duplicate publications were excluded. We excluded 101 studies for various reasons by evaluating the titles and abstracts.

**Figure 1:**
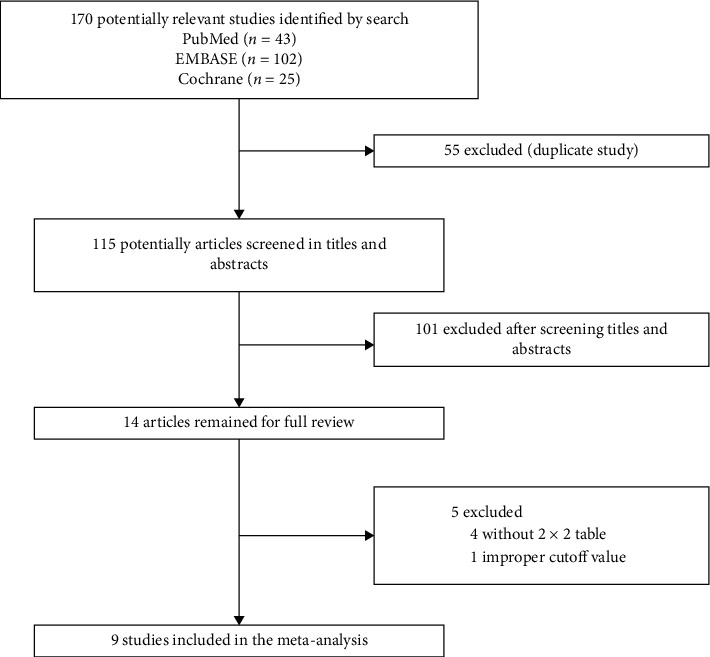
Flow diagram for the identification of eligible studies.

The remaining 14 articles were further scrutinized by reading the full text. Four studies were excluded due to an inability to extract a 2 × 2 contingency table [30–33], and one retrospective study was excluded after discussion between two authors because it used peak NT-proBNP as the cutoff value [34]. In total, nine studies [23–25, 35–40] including 2832 patients fulfilled the inclusion criteria and were ultimately included in this meta-analysis (Figure 1).

Table 1 summarizes the details of the nine included studies (prospective: 7; retrospective: 2). These studies were published between 2013 and 2020. Different SEN, SPE, and AUC values of BNP or NT-proBNP for the diagnosis of CI-AKI were reported. The AUC values in the studies ranged from 0.65 to 0.92, and the definition of CI-AKI varied. Five studies [23–25, 36, 37] measured NT-proBNP and four [35, 38–40] BNP. The decision cutoff value used in the studies varied widely between 42.4 and 676 pg/ml for BNP and between 512 and 2320 pg/ml for NT-proBNP (Table 2). Five studies included patients only with STEMI [24, 35, 37, 38, 40]. One study [37] was published in Russian, and the others were published in English.

### 3.2. Study Quality and Publication Bias

**Figure 2:**
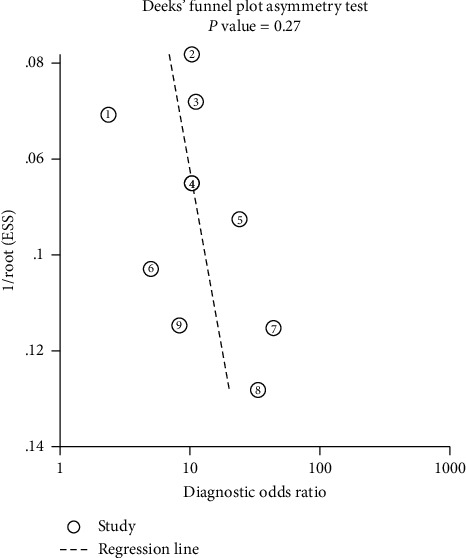
Deek funnel plot asymmetry test for publication bias, with *P* < 0.1 indicating publication bias. There was no significant publication bias (*P*=0.27).

Supplementary material S1 shows the risk of bias in the nine included studies. The results revealed that one study had a high risk of bias in the flow and timing domain [37]. Because that study initially included 103 patients, only 68 patients were assessed. A Deek funnel plot is shown in Figure 2. No significant publication bias was detected (*P*=0.27).

### 3.3. Diagnostic Value of Brain Natriuretic Peptide for CI-AKI Prediction

**Figure 3:**
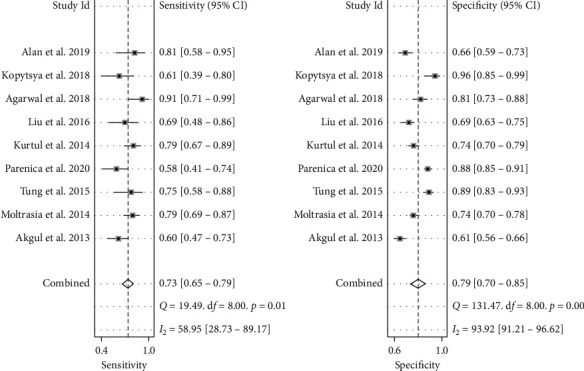
Forest plot of the sensitivity and specificity of brain natriuretic peptide for the diagnosis of contrast-induced acute kidney injury in patients with acute coronary syndrome undergoing coronary angiography. The pooled SEN and SPE values were 0.73 (95% CI 0.65–0.79) and 0.79 (95% CI 0.70–0.85), respectively. SEN, sensitivity and SPE, specificity.

**Figure 4:**
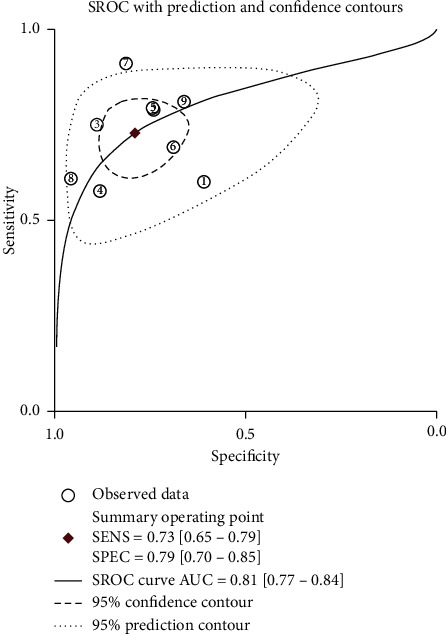
Summary receiver operating characteristic curve for the included studies. The AUC of brain natriuretic peptide for the diagnosis of contrast-induced acute kidney injury was 0.81 (95% CI 0.77–0.84), indicating a high diagnostic value. SROC, summary receiver operating characteristic; AUC, area under curve; SEN, sensitivity and SPE, specificity.

**Figure 5:**
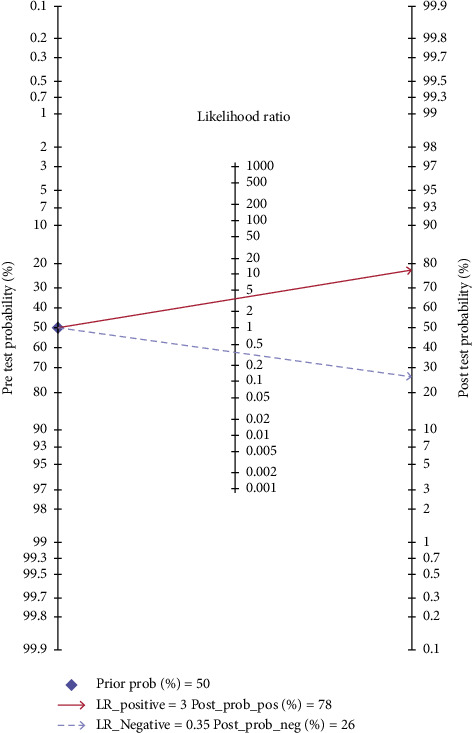
Fagan nomogram of brain natriuretic peptide for the diagnosis of contrast-induced acute kidney injury in patients with acute coronary syndrome undergoing coronary angiography. The pretest probability was set to 50%. The use of brain natriuretic peptide for the detection of contrast-induced acute kidney injury increased the posttest probability to 78% when the brain natriuretic peptide results were positive; the posttest probability decreased to 26% when the brain natriuretic peptide results were negative.

The pooled SEN and SPE values were 0.73 (95% CI 0.65–0.79) and 0.79 (95% CI 0.70–0.85), respectively (Figure 3). DOR was 10 (95% CI 6–17); PLR and NLR were 3.5 (95% CI 2.4–4.9) and 0.35 (95% CI 0.27–0.44), respectively (supplementary material S2). The SROC curve is depicted in Figure 4. The AUC of brain natriuretic peptide for the diagnosis of CI-AKI was 0.81 (95% CI 0.77–0.84), indicating a high diagnostic value. Based on the Fagan nomogram (Figure 5), if the pretest probability was set to 50%, the use of BNP or NT-proBNP for the detection of CI-AKI increased the posttest probability to 78% when the brain natriuretic peptide results were positive; the posttest probability decreased to 26% when the brain natriuretic peptide results were negative. The above results suggest that BNP or NT-proBNP is a useful biomarker for the diagnosis of CI-AKI in patients with ACS undergoing coronary angiography.

A total of 4 studies included patients diagnosed with ACS but not subdivided into UA, STEMI, or NSTEMI and were termed the “ACS” subgroup [23, 25, 36, 39]. Five others focusing on patients with STEMI used the term the “STEMI” subgroup [24, 35, 37, 38, 40]. Interestingly, brain natriuretic peptide showed a great diagnostic value in the “ACS” subgroup, with an estimated AUC of 0.85 (95% CI 0.81–0.88). Pooled SEN and SPE were 0.81 (95% CI 0.74–0.86, *I*^2^ = 0) and 0.74 (95% CI 0.69–0.78, *I*^2^ = 69.06%), respectively (Table 3).

### 3.4. Threshold Effect and Heterogeneity Analysis

The overall *I*^2^ value for the bivariate model was 90% (95% CI 81–100). The proportion of heterogeneity likely caused by the threshold effect was not significant (*P*=0.08). For the pooled SEN and SPE, the *I*^2^ values were 58.95% and 93.92%, respectively. Subgroup analysis based on the patient's condition (“STEMI” subgroup or “ACS” subgroup) revealed that heterogeneity in SEN may be caused by the patient's condition. The pooled *I*^2^ values for SEN in the “STEMI” subgroup and “ACS” subgroup were 9.1% and 0, respectively, though significant heterogeneity in SPE was observed. The results failed to show that different biomarkers or study types were the sources of the potential heterogeneity in SEN and SPE (Table 3).

## 4. Discussion

This is the first meta-analysis to evaluate the value of brain natriuretic peptide for CI-AKI in patients with ACS undergoing coronary angiography. Overall, the results suggested that BNP or NT-proBNP is a useful biomarker for the diagnosis of CI-AKI (AUC = 0.81, SEN = 0.73, and SPE = 0.79). The finding applies to both BNP (AUC = 0.78, SEN = 0.69, and SPE = 0.80) and NT-proBNP (AUC = 0.82, SEN = 0.77, and SPE = 0.78).

CI-AKI is a frequent complication in patients who receive iodinated contrast agent [41], and it is a common cause of hospital-acquired AKI and accounts for approximately 11% of hospital-acquired renal failure [42]. Previous studies have indicated that CI-AKI is associated with adverse clinical outcomes, including prolonged hospitalization, an increased risk of mortality, stent restenosis, and cardiovascular and cerebrovascular events in patients with ACS undergoing coronary angiography [43, 44]. At present, the diagnosis of CI-AKI is based on the increased serum creatinine concentration after a contrast agent injection. However, changes in serum creatinine lack sensitivity because in healthy people, nearly 50% of the glomerular filtration rate (GFR) must be lost before changes in serum creatinine can be detected [7, 45]. Moreover, there are no consistent thresholds of serum creatinine levels for the diagnosis of CI-AKI [41]. Thus, finding new biomarkers is of great significance for the early prediction of CI-AKI.

CI-AKI in ACS is a multifactorial phenomenon. First, the contrast agent is completely excreted by the kidney, and the concentration of the contrast agent increases as it passes through the renal tubules, possibly reaching a level toxic to tubular cells [41]. Toxins can have direct cytotoxic effects on endothelial cells or renal tubular epithelial cells, impair renal hemodynamics, and lead to the precipitation of metabolites or crystals, among others [7]. In addition, the impaired cardiac output and increased venous congestion lead to systemic and renal hemodynamic changes, which result in a decrease in GFR. This may be a key mechanism in the pathogenesis of AKI. Moreover, patients with ACS are characterized by progressive activation of several neurohormonal systems, involving an imbalance of endogenous vasodilating and vasoconstrictive factors and exerting profound effects on kidney perfusion and function [46]. Although the mechanism underlying the relationship between brain natriuretic peptide and CI-AKI has not been explained completely, the following reasons might be involved it to some extent. First, renal hemodynamic impairment in the context of ACS may decrease the clearance of brain natriuretic peptide [47]. Second, Vila et al. found that in healthy people with normal heart function, plasma brain natriuretic peptides were elevated in a model of systemic inflammation [48], and brain natriuretic peptide is accepted as an acute-phase reactant [49]. Therefore, brain natriuretic peptide may be an indicator of increased inflammation and immune response in ACS, which plays an important role in the occurrence and development of CI-AKI [50, 51]. Furthermore, AKI primarily presents as a sharp decrease in GFR and water and sodium retention, which accelerate the overall progression of cardiovascular disease and heart failure, followed by an increase in BNP or NT-proBNP [52]. Nonetheless, more research are needed to identify the potential mechanism between brain natriuretic peptides and CI-AKI.

BNP or NT-proBNP elevation in AKI patients was found in clinical practice, and recent data suggest that baseline BNP or NT-proBNP may help to identify ACS patients at risk for CI-AKI after coronary angiography. The results from our meta-analysis confirm the role of BNP or NT-proBNP in predicting CI-AKI. Furthermore, we built a Fagan nomogram and a likelihood ratio scattergram to evaluate the clinical application value.

There was considerable heterogeneity among the included studies. Although we conducted sensitivity and subgroup analyses, the heterogeneity was not significantly decreased. This may be caused by different cutoff values, different definitions of CI-AKI, different conditions of patients, or different study designs among the included studies. Some studies measured NT-proBNP, whereas others measured BNP, and the number of participants in the different studies varied greatly, which may also lead to heterogeneity. More high-quality studies are required to shed light on the role of brain natriuretic peptide in the diagnosis of CI-AKI for ACS patients undergoing coronary angiography.

Measuring brain natriuretic peptide is inexpensive, repeatable, and easy to achieve. For patients with ACS, monitoring brain natriuretic peptide is important and essential. Combining brain natriuretic peptide, creatinine, urine output, and other novel biomarkers, such as neutrophil gelatinase-associated lipocalin (NGAL) and cystatin C, which have been identified as potential biomarkers of CI-AKI [53, 54], can improve early diagnostic precision for CI-AKI. Moreover, early detection, intervention, and treatment contribute to a favorable prognosis in CI-AKI.

There are several limitations in our meta-analysis. First, the definition of CI-AKI was not completely consistent among the included studies. Second, in terms of sample size, brain natriuretic peptide assays, cutoff values, and study type differed across the included studies, which may have led to heterogeneity. Finally, substantial heterogeneity existed, and additional subgroup analyses could not be performed to reduce and interpret the heterogeneity because a limited number of studies were included in the meta-analysis.

## 5. Conclusions

This study is the first meta-analysis to evaluate the diagnostic value of brain natriuretic peptide for CI-AKI in patients with ACS undergoing coronary angiography, and the results suggest that BNP or NT-proBNP can serve as an effective predictive marker for CI-AKI. However, additional high-quality studies are required to find the optimal cutoff value and the diagnostic value of BNP or NT-proBNP in combination with other biomarkers.

## Figures and Tables

**Table 1:** Characteristics of included studies.

Marker	Study	Study type	Definition of CI-AKI	Population	No. of patients	Male/female	Mean age
BNP	Akgul et al. 2013 [40]	Prospective	An absolute increase in SCr of ≥0.3 mg/dl or ≥50% from the baseline	STEMI patients undergoing PCI	424	340/84	55.3 ± 12.0
	Moltrasio et al. 2014 [39]	Prospective	An absolute increase in SCr of ≥0.3 mg/dl	ACS patients undergoing PCI	639	484/155	70.6 ± 12.5
	Tung et al. 2015 [38]	Prospective	An absolute increase in SCr of ≥0.3 mg/dl or ≥50% from the baseline	STEMI patients undergoing PCI	189	163/26	62.6 ± 13.9
	Parenica et al. 2020 [35]	Retrospective	An absolute increase in SCr of ≥0.3 mg/dl or ≥50% from the baseline	STEMI patients undergoing PCI	427	328/99	73 (45–83)*∗* 62 (45–78)*∗*

NT-proBNP	Kurtul et al. 2014 [25]	Prospective	An increase in SCr of ≥0.5 mg/dL or ≥25% above baseline within 72 hours after contrast administration	ACS patients undergoing PCI	436	280/156	62.27 ± 13.01
	Liu et al. 2016 [24]	Prospective	An increase in SCr of >0.5 mg/dL above baseline within 48 to 72 hours after contrast administration	STEMI patients undergoing PCI	283	NA	62.9 ± 12.3
	Agarwal et al. 2018 [23]	Prospective	An increase in SCr of ≥0.5 mg/dL or ≥25% above baseline within 48 hours after index angiography	ACS patients undergoing PCI	150	96/54	63.03 ± 9.07
	Kopytsya et al. 2018 [37]	Retrospective	An absolute increase in SCr of ≥0.3 mg/dl from the baseline within 48 hours	STEMI patients undergoing SCAG	68	NA	NA
	Alan et al. 2019 [36]	Prospective	An absolute increase in SCr of ≥0.3 mg/dl at 48 h of injection or >50% above baseline within 72 hours after contrast administration	ACS patients undergoing coronary angiography	216	170/46	63.9 ± 12.3

BNP, B-type natriuretic peptide; NT-proBNP, N-terminal pro-B-type natriuretic peptide; CI-AKI, contrast-induced acute kidney injury; SCr, serum creatinine; ACS, acute coronary syndrome; STEMI, ST-elevation myocardial infarction; PCI, percutaneous coronary intervention; SCAG, selective coronary angiography; NA, not available; *∗*median (5th–95th percentile ranges).

**Table 2:** BNP and NT-proBNP measurements.

Marker	Study	Assay	Optimal timing	Cutoff (pg/ml)	AUC	SEN/SPE, %	TP/FP/TN/FN
BNP	Akgul et al. 2013 [40]	Biosite triage meter	On admission	42.4	0.65	60/61	35/143/223/23
	Moltrasio et al. 2014 [39]	Beckman coulter, triage	On admission	184	0.7	79/74	67/144/410/18
	Tung et al. 2015 [38]	Biosite diagnostics, triage	On admission	676	0.86	75/89	27/17/136/9
	Parenica et al. 2020 [35]	Enzyme immunoassay, (abbott laboratories)	12 h after admission	623	0.75	57.9/88.2	22/46/343/16

NT-proBNP	Kurtul et al. 2014 [25]	Elecsys 2010 analyzer, (roche diagnostics)	Before angiography	2149	0.83	79.4/74.3	50/96/277/13
	Liu et al. 2016 [24]	Electrochemiluminescence immunoassay, (roche diagnostics)	On admission	1800	0.76	69/70	18/80/178/8
	Agarwal 2018 [23]	NA	On admission	2320	0.92	90.9/81.5	20/24/104/2
	Kopytsya et al. 2018 [37]	Enzyme-like immunoassay	At the 1st day of STEMI.	1345	0.75	61.5/94.9	14/2/43/9
	Alan et al. 2019 [36]	NA	NA	512	0.79	81/66	17/66/129/4

BNP, B-type natriuretic peptide; NT-proBNP, N-terminal pro-B-type natriuretic peptide; STEMI, ST-elevation myocardial infarction; AUC, area under curve; SEN, sensitivity; SPE, specificity; TP, true positives; TN, true negatives; FP, false positives; FN, false negatives and NA, not available.

**Table 3:** Results of sensitivity analysis and subgroup analysis.

Categories	Number of studies	Sensitivity (95% CI)/*I*^2^	Specificity (95% CI)/*I*^2^	AUC (95% CI)	DOR (95% CI)	PLR/NLR
All studies	9 [23–25, 35–40]	0.73 (0.65, 0.79)/58.95	0.79 (0.70, 0.85)/93.92	0.81 (0.77, 0.84)	10 (6, 17)	3.5/0.35

*Biomarker*						
BNP	4 [35, 38–40]	0.69 (0.59, 0.78)/72.06	0.80 (0.67, 0.89)/97.22	0.78 (0.75, 0.82)	9 (4, 20)	3.4/0.39
NT-proBNP	5 [23–25, 36, 37]	0.77 (0.68, 0.83)/43.57	0.78 (0.66, 0.87)/84.33	0.82 (0.79, 0.85)	12 (7, 21)	3.5/0.30

*Patient's condition*						
STEMI	5 [24, 35, 37, 38, 40]	0.64 (0.57, 0.71)/9.1	0.83 (0.69, 0.92)/97.08	0.66 (0.62, 0.70)	9 (4, 21)	3.8/0.43
ACS	4 [23, 25, 36, 39]	0.81 (0.74, 0.86)/0	0.74 (0.69, 0.78)/69.06	0.85 (0.81, 0.88)	12 (7, 20)	3.1/0.26
Prospective study	7 [23–25, 36, 38–40]	0.76 (0.69, 0.82)/52.08	0.74 (0.67, 0.80)/90.41	0.82 (0.78, 0.85)	9 (5, 17)	2.9/0.32
Undergoing PCI	7 [23–25, 35, 38–40]	0.73 (0.65, 0.80)/65.65	0.78 (0.70, 0.84)/94.60	0.82 (0.78, 0.85)	10 (5, 17)	3.3/0.34

BNP, B-type natriuretic peptide; NT-proBNP, N-terminal pro-B-type natriuretic peptide; ACS, acute coronary syndrome; STEMI, ST-elevation myocardial infarction; PCI, percutaneous coronary intervention; AUC, area under curve; PLR, positive likelihood ratio; NLR, negative likelihood ratio; DOR, diagnostic odds ratio and CI, credible interval.

## Data Availability

The data used to support the findings of this study are included within the article and its supplementary material files.

## List of abbreviations

ACS: Acute coronary syndrome
AKI: Acute kidney injury
AUC: Area under curve
BNP: B-type natriuretic peptide
CI-AKI: Contrast-induced acute kidney injury
CI: Credible interval
DOR: Diagnostic odds ratio
FN: False negatives
FP: False positives
NT-proBNP: N-terminal pro-B-type natriuretic peptide
NLR: Negative likelihood ratio
NSTEMI: Non-ST elevation myocardial infarction
PLR: Positive likelihood ratio
PCI: Percutaneous coronary intervention
PRISMA: Preferred Reporting Items for Systematic Reviews and Meta-Analyses
QUADAS-2: Quality Assessment of Diagnostic Accuracy Studies-2
SEN: Sensitivity
STEMI: ST-elevation myocardial infarction
SPE: Specificity
SROC: Summary receiver operating characteristic
TP: True positives
TN: True negatives
UA: Unstable angina.

## Supplementary Materials

Supplementary Materials Supplementary material S1: summary of the methodological quality of the studies according to the QUADAS-2 (Quality Assessment of Diagnostic Accuracy Studies-2) criteria. Supplementary material S2: likelihood ratio scattergram.
