# Supplementary material for: Brain Natriuretic Peptide for Predicting Contrast-Induced Acute Kidney Injury in Patients with Acute Coronary Syndrome Undergoing Coronary Angiography: A Systematic Review and Meta-Analysis
Source: J Interv Cardiol. 2020 Sep 19;2020:1035089. doi: 10.1155/2020/1035089 (PMC7520681; doi:10.1155/2020/1035089)
Supplement: Supplementary Materials — Supplementary material S1: summary of the methodological quality of the studies according to the QUADAS-2 (Quality Assessment of Diagnostic Accuracy Studies-2) criteria. Supplementary material S2: likelihood ratio scattergram. [file 1035089.f1.docx]

**Supplementary material S1:** Summary of the methodological quality of the studies according to the QUADAS-2 criteria.

QUADAS-2, Quality Assessment of Diagnostic Accuracy Studies-2.


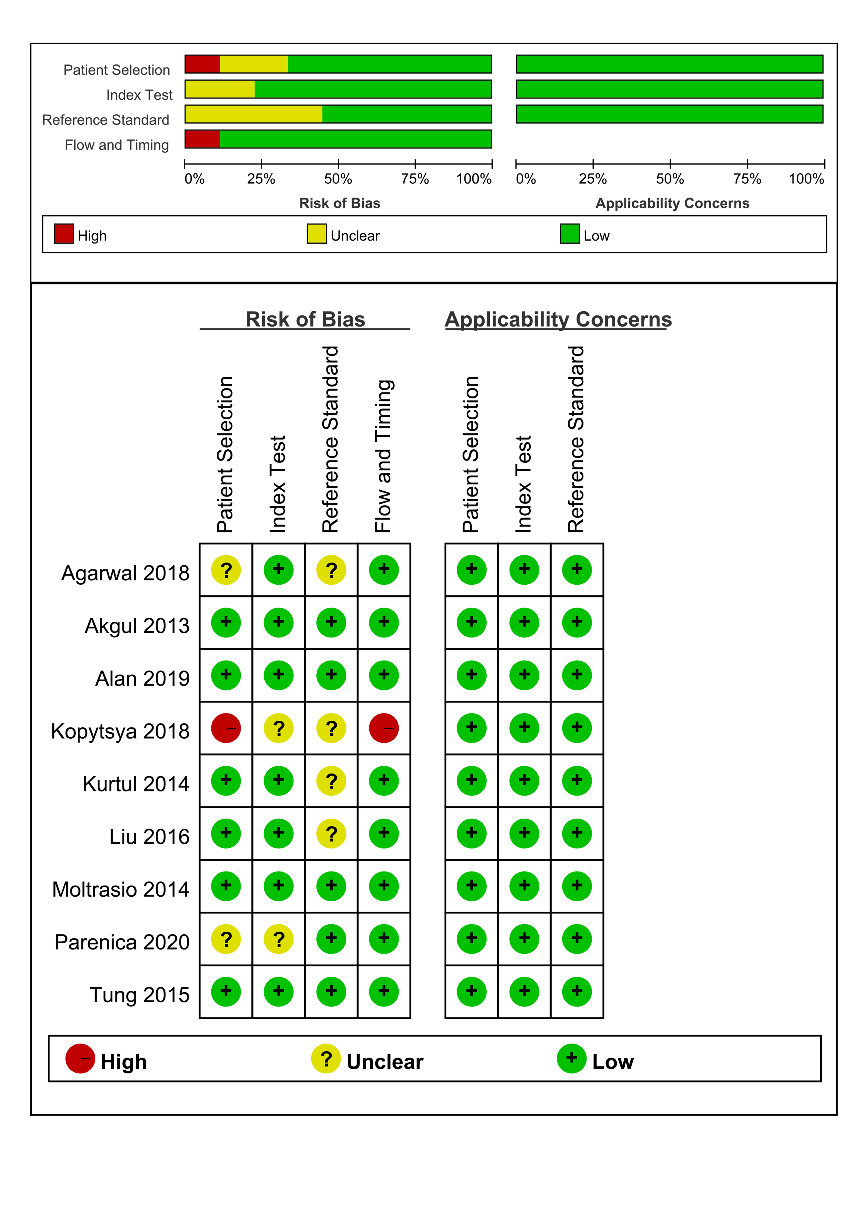


**Supplementary material S2:** Likelihood ratio scattergram.


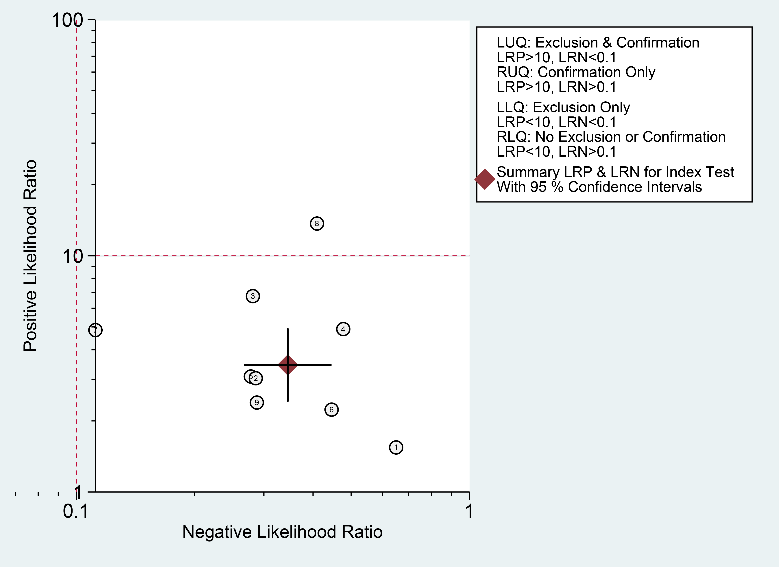


Likelihood ratio scattergram of brain natriuretic peptide for the diagnosis of contrast-induced acute kidney injury. The positive likelihood ratio and negative likelihood ratio were 3.5 and 0.35, respectively.

LLQ, left lower quadrant; LRN, likelihood ratio negative; LRP, likelihood ratio positive; LUQ, left upper

Quadrant; RLQ, right lower quadrant; RUQ, right upper quadrant.
